# Multi-Scale Image Defogging Network Based on Cauchy Inverse Cumulative Function Hybrid Distribution Deformation Convolution

**DOI:** 10.3390/s25165088

**Published:** 2025-08-15

**Authors:** Lu Ji, Chao Chen

**Affiliations:** College of Aeronautics, Nanjing University of Aeronautics and Astronautics, Nanjing 210006, China

**Keywords:** image defogging, Cauchy distribution, deformable convolution, attention mechanism, inverse Cauchy integral function

## Abstract

The aim of this study was to address the issue of significant performance degradation in existing defogging algorithms under extreme fog conditions. Traditional Taylor series-based deformable convolutions are limited by local approximation errors, while the heavy-tailed characteristics of the Cauchy distribution can more successfully model outliers in fog images. The following improvements are made: (1) A displacement generator based on the inverse cumulative distribution function (ICDF) of the Cauchy distribution is designed to transform uniform noise into sampling points with a long-tailed distribution. A novel double-peak Cauchy ICDF is proposed to dynamically balance the heavy-tailed characteristics of the Cauchy ICDF, enhancing the modeling capability for sudden changes in fog concentration. (2) An innovative Cauchy–Gaussian fusion module is proposed to dynamically learn and generate hybrid coefficients, combining the complementary advantages of the two distributions to dynamically balance the representation of smooth regions and edge details. (3) Tree-based multi-path and cross-resolution feature aggregation is introduced, achieving local–global feature adaptive fusion through adjustable window sizes (3/5/7/11) for parallel paths. Experiments on the RESIDE dataset demonstrate that the proposed method achieves a 2.26 dB improvement in the peak signal-to-noise ratio compared to that obtained with the TaylorV2 expansion attention mechanism, with an improvement of 0.88 dB in heavily hazy regions (fog concentration > 0.8). Ablation studies validate the effectiveness of Cauchy distribution convolution in handling dense fog and conventional lighting conditions. This study provides a new theoretical perspective for modeling in computer vision tasks, introducing a novel attention mechanism and multi-path encoding approach.

## 1. Introduction

Single-image defogging, as a highly challenging image restoration task in computer vision, aims to reconstruct clear scene radiations from foggy observation images. Early methods based on physical priors, such as atmospheric scattering models [1] and dark channel priors [2], achieved promising preliminary results but were limited due to simplifications of assumptions about complex atmospheric conditions. With the rise of deep learning, Convolutional Neural Network (CNN) [3,4,5] architectures have gradually pushed the boundaries of dehazing performance through technological innovations such as multi-scale fusion [6], deformable convolutions [7], and attention mechanisms [8]. Recent advances in feature extraction architectures, including Multiscale DenseNet with bidirectional recurrent networks (Bi-RNN) [9] and graph-based approaches like the Neighborhood Adaptive Graph Isomorphism Network [10], have demonstrated remarkable success in handling complex spatial–spectral relationships, offering valuable insights for fog density estimation in defogging tasks. However, existing methods still face two core challenges when handling non-uniform haze (e.g., strong fog lines or regions with abrupt depth changes): (1) the fixed sampling grid of traditional convolutions struggles to adapt to the spatial heterogeneity of fog concentration; (2) the quadratic computational complexity of standard self-attention models limits their application in pixel-level dense prediction tasks.

Recently, breakthroughs in the performance of visual self-attention models [11] in high-level visual tasks has led to explorations of their application in fog removal. However, direct transplantation faces two challenges: first, the original self-attention mechanism has a spatial complexity of O (n^2^), and even window partitioning strategies [12] sacrifice global context modeling capabilities; second, existing visual self-attention models [13] typically use fixed-size convolution kernels to generate feature units, which cannot flexibly adapt to the dynamic distribution of multi-scale features in foggy images. An improved method for the traditional Retinex algorithm [14] was proposed by constructing a single-parameter homomorphic filter function using the Sigmoid function, effectively correcting the lighting in foggy images. However, due to the Retinex algorithm’s limitations, improvements in its performance were still limited. Yang, P, W [15] propose a lightweight image defogging method that combines pixel and frequency domain information by improving the upsampling and downsampling mechanisms through discrete wavelet transform and utilizing frequency domain features for auxiliary processing, achieving an outstanding performance of 41.11 dB on the SOTS-Indoor dataset. The Multi-Branch Taylor Transformer V2 (MB-TaylorFormerV2) [16,17] linearizes self-attention through Taylor expansion, partially alleviating the computational bottleneck. However, there is still room for improvement in terms of approximation error compensation and local–global feature synergy.

In response to the aforementioned challenges, the technical contributions of this paper can be summarized as follows: (1) For the first time, the inverse Cauchy cumulative distribution function is used in the offset generation process of deformation convolution, providing a mathematical foundation for handling non-uniform haze. A novel double-peak inverse Cauchy cumulative function is proposed, which, compared to the single-peak function, not only exhibits the advantage of heavy tails but also explicitly models multi-modal offsets, significantly enhancing the method’s expressive capability in complex deformation scenarios. (2) An innovative hybrid Cauchy–High-Speed distribution self-attention linearization method is proposed, achieving a performance similar to the original self-attention model while maintaining O (n) complexity. Innovative dynamically learning hybrid coefficients based on fog concentration are proposed, combining the complementary advantages of the two distributions to dynamically balance the representation of smooth regions and edge details. (3) A multi-path collaborative tree-based processing pipeline is designed to achieve adaptive extraction and fusion of multi-scale features. Through the above novel and improved strategies, efficient and high-quality defogging capabilities are achieved.

## 2. Theory and Methods

### 2.1. Cauchy and Inverse Convolution Deformation Model

#### 2.1.1. Mathematical Foundations

The core limitations of traditional CNN convolution kernels lie in their rigid locality constraints: 1. Spatial Restrictions: their fixed grid sampling (e.g., 3 × 3) only captures neighborhood interactions, failing to model large-scale geometric deformations (e.g., rotation and perspective), leading to artifacts in depth discontinuity regions of foggy images. 2. Single-Scale Processing: each convolutional kernel operates on a fixed scale, requiring manually designed multi-scale architectures to increase complexity. 3. Channel Isolation: independent computation across channels ignores spectral/semantic correlations, limiting accuracy in tasks like hyperspectral classification. The Cauchy convolution kernel overcomes these limitations through probabilistic deformation modeling: 1. Heavy-Tailed Offsets: this generates continuous spatial offsets (e.g., ±15 pixels) via the Cauchy inverse CDF, supporting extreme deformations like 60° rotation. 2. Dynamic Multi-Scale Adaptation: the scale parameter adaptively adjusts the sampling density, enabling multi-scale feature processing in a single layer. 3. Cross-Channel Collaboration: the offset generation network implicitly learns inter-channel dependencies and upgrades discrete linear transformations into a probability-driven continuous geometry processor.

The Cauchy inverse cumulative distribution function [18] is integrated into the offset generation process of deformable convolutions. The long-tail characteristics of the Cauchy distribution are utilized to enhance the model’s ability to model extreme deformations such as large rotations and perspective transformations, while adaptive adjustment is achieved through distribution parameters.

The probability density function of the Cauchy distribution is calculated as follows:

(1) $$f\left(x;x_{0},\gamma \right)=\frac{1}{\pi \gamma \left[1+{\left(\frac{x-x_{0}}{\gamma }\right)}^{2}\right]}$$

where $x_{0}$ is the position parameter, which controls peak position; γ is the scale parameter, which controls the distribution width.

The Cauchy inverse cumulative distribution function is as follows:

(2) $$F^{-1}\left(p;x_{0},\gamma \right)=x_{0}+\gamma \cdot tan(\pi (p-0.5))$$

where p∈(0,1) is the uniformly distributed probability values, with the output being random variables obeying the Cauchy distribution, with heavy-tail characteristics.

The advantages of this are as follows:

1. Polynomial decay is slower.

At $x\to \infty ,f(x)\approx \frac{1}{\pi \gamma }\cdot \frac{1}{(x/\gamma {)}^{2}}=\frac{\gamma }{\pi x^{2}}∼O\left(x^{-2}\right)$, and the exponential decay is slower than that of the Gaussian distribution. At x=5γ, f(x) value $f(5\gamma )=\frac{1}{26\pi \gamma }\approx 0.012f(0)$, with a significant probability density (compared to that of Gaussian distribution, which is only $2.9\times 10^{-7}f(0$)).

2. Better dimensional flexibility.

The distribution shape is dynamically adjusted through learnable parameters.

A small γ<0.4 is suitable for fine texture restoration and high-precision edge detection; a large γ>0.6, with a flat distribution and a flat heavy tail, is suitable for large-scale fog line tracking.

3. Better gradient stability.

The Cauchy distribution gradient expression is as follows:

(3) $$\nabla_{x}f(x)=-\frac{2\left(x-x_{0}\right)}{\pi \gamma^{3}{\left[1+{\left(\frac{x-x_{0}}{\gamma }\right)}^{2}\right]}^{2}}$$

where $\nabla_{x}f(x$), the maximum value appears at $x=x_{0}\pm \frac{\gamma }{\sqrt{3}}$, and the amplitude is always less than ${\left\|\nabla_{x}f\right\|}_{max}=\frac{3\sqrt{3}}{8\pi \gamma^{2}}$. This avoids the problem of gradient vanishing when x→∞.

#### 2.1.2. Innovative Solutions

The traditional single-peak Cauchy is extended to a double-peak form as follows:

(4) $$f\left(x;x_{0}^{1},x_{0}^{2},\gamma_{1},\gamma_{2,}w\right)=w\cdot \frac{1}{\pi \gamma_{1}\left[1+{\left(\frac{x-x_{0}^{1}}{\gamma_{1}}\right)}^{2}\right]}+(1-w)\cdot \frac{1}{\pi \gamma_{2}\left[1+{\left(\frac{x-x_{0}^{2}}{\gamma_{2}}\right)}^{2}\right]}$$

where $x_{0}^{1},x_{0}^{2}$ is a double peak position, which is a learnable parameter; $\left\|x_{0}^{1}-x_{0}^{2}\right\|$ controls the peak spacing; $\gamma_{1},\gamma_{2}$ is the scale parameter, which controls the degree of overlap between the two peaks; and w∈[0,1] is the mode weight (dynamically predicted by the input features).

The sampling of the inverse cumulative distribution function of Cauchy distribution is improved by achieving double-peak sampling through mixed probability:

(5) $$F^{-1}(p;x_{0}^{1},x_{0}^{2},\gamma_{1},\gamma_{2,}w)=w\cdot \left[x_{0}^{1}+\gamma_{1}tan(\pi (p-0.5))\right]+(1-w)\cdot \left[x_{0}^{2}+\gamma_{2}tan(\pi (p-0.5))\right]$$

**Figure 1:**
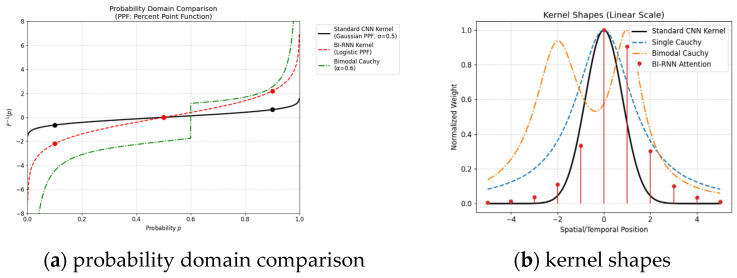
Double-peak inverse cumulative distribution functions of Cauchy distribution.

The bimodal Cauchy distribution retains the advantages of a single peak and heavy tail while incorporating a dynamic shift mechanism. The main peak $x_{0}^{1}$ models the physical scattering of fog layers, while the secondary peak $x_{0}^{2}$ captures local fog concentration fluctuations and fog penetration at building edges. The adaptive weight w is dynamically adjusted through a fog concentration weight sensing module. By explicitly modeling multi-modal shifts, the method significantly improves its expressive capability in complex deformable scenes. As shown in Figure 1a, the bimodal Cauchy kernel excels due to its dual-peak heavy-tailed properties: Multimodal Modeling: With α = 0.6 (green dash-dot line in the plot shows dual inflection points at *p* ≈ 0.2 and 0.8), it captures two distinct feature patterns simultaneously, ideal for complex data (e.g., multi-object detection). Heavy-Tail Robustness: Unlike the Gaussian PPF (black line) with steep tail decay, the bimodal Cauchy’s F^−1^ (p) declines gently (e.g., F^−1^ (0.001) ≈ −4), enhancing sensitivity to outliers/extreme events. Adaptability: Adjusting α controls peak separation, balancing local feature extraction and long-range dependency modeling. As shown in Figure 1b, (1) double-peak feature extraction (orange dotted line in the figure) simultaneously captures local and mid-range features through the main peak (0 position) and secondary peak (+2 position), making it particularly suitable for processing periodic structures or symmetrical targets; (2) Controllable non-locality, with a valley design in the −2 to +1 interval to avoid feature blurring, expanding the receptive field while maintaining position sensitivity; (3) Adaptive weight balancing: The normalized weight of 0.6 for the double peak outperforms the smooth distribution of the single-peak Cauchy (blue dashed line), and its adjustable α parameter (α=0.6 in the figure) can flexibly adapt to feature detection requirements at different scales.

#### 2.1.3. Operating Network Design

Given an input feature map $X\in R^{B\times C\times H\times W}$, the offset generation process of Cauchy deformation convolution consists of three steps, where B is the batch size, which is the number of samples processed simultaneously; C is the number of channels, indicating the depth of the feature map; H is the height (number of pixels) of an image or feature map; and W is the width (number of pixels) of an image or feature map:

1. Basic offset: the initial coarse offset is obtained by sampling through the inverse convolution function:
  (6) $$F_{base}^{-1}\left(p;x_{0}^{1},x_{0}^{2},\gamma_{1},\gamma_{2,}w\right)=w\cdot \left[x_{0}^{1}+\gamma_{1}tan(\pi (p-0.5))\right]+(1-w)\cdot \left[x_{0}^{2}+\gamma_{2}tan(\pi (p-0.5))\right]$$
2. Adaptive correction: a lightweight CNN with 3/5/7 layers (number of parameters < 1 k) is used to predict pixel-wise correction coefficients.
  (7) $$F_{\mathrm{final}}^{-1}\left(p;x_{0}^{1},x_{0}^{2},\gamma_{1},\gamma_{2,}w\right)=F_{base}^{-1}\left(p;x_{0}^{1},x_{0}^{2},\gamma_{1},\gamma_{2,}w\right)⊙(1+\alpha )+\beta$$
  where α,β are predicted by the correction network and constrained within the range [−0.5,0.5], α adjusts the amplitude of the offset and enhances adaptability to high-frequency details, β compensates for system bias and solves the center offset problem caused by the symmetry of the Cauchy distribution; and ⊙ denotes element-wise multiplication.
3. Design of adaptive truncation strategy constraints.

To ensure training stability and prevent numerical explosion of the tan (·) function when p→0/1, controllable deformation is performed, with a threshold τ and double-peak scale $\gamma_{1},\gamma_{2}$ parameters that are dynamically associated to ensure that, in low fog concentration regions (γ↓), larger deformation exploration is allowed, and in high fog concentration regions (γ↑), constraints are strengthened to avoid overfitting. Therefore, an adaptive truncation strategy is designed:

(8) $$F_{\mathrm{clipped}}^{-1}\left(p;x_{0}^{1},x_{0}^{2},\gamma_{1},\gamma_{2,}w\right)=\left\{\begin{array}{ll} F_{\mathrm{final}}^{-1}\left(p;x_{0}^{1},x_{0}^{2},\gamma_{1},\gamma_{2,}w\right) & {∥F_{\mathrm{final}}^{-1}\left(p;x_{0}^{1},x_{0}^{2},\gamma_{1},\gamma_{2,}w\right)∥}_{2}\leq \tau \\ \tau \frac{F_{\mathrm{final}}^{-1}\left(p;x_{0}^{1},x_{0}^{2},\gamma_{1},\gamma_{2,}w\right)}{{∥F_{\mathrm{final}}^{-1}\left(p;x_{0}^{1},x_{0}^{2},\gamma_{1},\gamma_{2,}w\right)∥}_{2}} & \mathrm{otherwise} \end{array}\right.$$

where the threshold $\tau =2(w\cdot \gamma_{1}+\left(1-w\right){\cdot \gamma }_{2}$) is dynamically adjusted during training. The L2 norm of the output $F^{-1}\left(p;x_{0}^{1},x_{0}^{2},\gamma_{1},\gamma_{2},w\right)$ of the inverse cumulative distribution function is calculated as follows: ${\left\|F^{-1}\left(p;x_{0}^{1},x_{0}^{2},\gamma_{1},\gamma_{2},w\right)\right\|}_{2}=\sqrt{{\left(\begin{array}{c} w\cdot \left[x_{0}^{1}+\gamma_{1}\tan\left(\pi \left(p-0.5\right)\right)\right]+(1-w)\cdot [x_{0}^{2}+\gamma_{2}\;\tan(\pi (p-0.5))] \end{array}\right)}^{2}}$.

### 2.2. Theory and Implementation of the Cauchy–Gaussian Hybrid Distribution Attention Mechanism

#### 2.2.1. Mathematical Foundations

The hybrid distribution attention mechanism adopts a dual-branch parallel structure, which dynamically fuses global context and local detail features through weight fusion:

(9) $${\mathrm{Attention}}_{\mathrm{hybrid}}=\lambda \cdot {\mathrm{Attention}}_{\mathrm{Cauchy}\;}+(1-\lambda )\cdot {\mathrm{Attention}}_{\mathrm{Gauss}}$$

where $\lambda \in \left[0,1\right]$ is a learnable hybrid coefficient, which is adaptively adjusted through Formula (19).

This design addresses the specific requirements of the fog removal task: 1. The Cauchy branch models long-range dependencies to address the discontinuity of the fog concentration spatial distribution. 2. The Gaussian branch captures local texture details to maintain edge sharpness. 3. Dynamic fusion automatically adjusts weights based on the fog concentration distribution.

#### 2.2.2. Innovative Cauchy Attention Branch

(1) Linearization Implementation Method

By approximating the Cauchy kernel function using a Taylor series expansion [19], the original $O\left(n^{2}\right)$ complexity is reduced to O(n):

(10) $$K_{\mathrm{Cauchy}}\left(x_{i},x_{j}\right)=\frac{1}{1+\frac{{∥x_{i}-x_{j}∥}^{2}}{\eta^{2}}}\approx \sum_{k=0}^{m}\;\frac{(-1{)}^{k}}{\eta^{2k}}{∥x_{i}-x_{j}∥}^{2k}$$

where m is the Taylor expansion order, balancing accuracy and efficiency; when m=2, the associative property of matrix multiplication is used to achieve linear computation:

(11) $${\mathrm{Attention}}_{\mathrm{Cauchy}}=Softmax\left(\frac{Q\left(K^{T}K\right)Q^{T}}{\eta^{4}}-\frac{2QK^{T}}{\eta^{2}}+1\right)\cdot V$$

where η is the scale parameter, controlling the attention decay rate, with an initial value of 0.5; $Q\in R^{B\times h\times H\times W\times d}$ is the query matrix, representing the features of the target location; $K\in R^{B\times h\times H\times W\times d}$ is the key matrix, representing the features of the source positions; $V\in R^{B\times h\times H\times W\times d}$ is the value matrix, representing the feature information to be aggregated; $Softmax(x)=\frac{e^{x_{i}}}{\sum_{i}\;e^{x_{i}}}$ is the activation function used for multi-class classification problems.

$\frac{Q\left(K^{T}K\right)Q^{T}}{\eta^{4}}$ captures the global second-order statistics of the feature space, enhancing the model’s ability to perceive complex patterns; $-\frac{2QK^{T}}{\eta^{2}}$ models local linear correlations, with the negative sign ensuring complementarity with the higher-order terms; +1 prevents the kernel function values from becoming too small, which could cause the gradients to vanish.

The value of η varies as shown in Equation (12):

(12) $$\eta =Softplus\left(w_{\eta }^{T}Pool(Q+K)+b_{\eta }\right)$$

where $w_{\eta }^{T}\in R^{h\times d}$ is the weight vector; d is the feature dimension of each attention head, where d=C/hC is the total number of channels; h is the number of attention heads, initialized as a Xavier normal distribution $N(0,\sqrt{1/d}$) and which performs a linear projection on the pooled features; $Pool\;(Q+K)\in R^{B\times h\times H\times W\times d}$ is the global average pooling function $\frac{1}{L}\sum_{i=1}^{L}\;\left(Q_{i}+K_{i}\right);b_{\eta }$ is the bias term, adjusting the baseline value of the parameters, and is a scalar; and $Softplus\in R^{B\times h}$ is the activation function ln (1 + $e^{x}$).

(2) Gradient constraint conditions

The upper bound of the Cauchy kernel gradient is as follows:

(13) $$∥\nabla_{\eta }K_{\mathrm{Cauchy}}∥\leq \frac{2}{\eta^{3}}\sum_{k=1}^{m}\;k{\left(1-\frac{1}{\eta^{2}}\right)}^{k-1}$$

When η>1, this ensures that the gradient is bounded and avoids training oscillations.

Stability condition:

(14) $$\|\nabla_{\eta }\mathrm{Attention}\;\left\|\leq \frac{4}{\eta^{3}}\right\|Q\left\|\|K\|+\frac{4}{\eta^{5}}\right\|Q\left\|\left\|K^{T}K\right\|\right.$$

#### 2.2.3. Innovative Gaussian Attention Branch

(1) Multi-Scale Modulation Mechanism

A pyramid attention mechanism is constructed using a three-level Gaussian kernel [20] (σ = 1, 3, 5):

(15) $${\mathrm{Attention}}_{\mathrm{Gauss}}=\sum_{s\in \{1,3,5\}}\;w_{s}\cdot Softmax\;\left(\frac{QK^{T}}{\sigma_{s}\sqrt{d_{k}}}\right)V$$

where $w_{s}$ is the mixed weight of the s-th level Gaussian kernel, which is dynamically generated by the input features, where $\sum w_{s}=1$.

A novel gradient-aware weight allocation parameter is dynamically generated and introduced [21]:

(16) $$w_{s}=Sigmoid\;(MLP\;(avgpool\;(Q),\|\nabla Q\|))$$

where avgpool (Q) is the global average pooling of the query matrix $avgpool(Q)=\frac{1}{L}\sum_{i=1}^{L}\;Q_{i};\|\nabla Q\|$ is the gradient magnitude of the query matrix $\left\|\nabla Q\right\|=\sqrt{(\partial Q/\partial x{)}^{2}\;+\;(\partial Q/\partial y{)}^{2}};\;MLP\left(\cdot \right)$ is a multi-layer perceptron that maps the pooled features to the scale weight space $R^{2d}\to R^{3};Sigmoid$ compresses the MLP output to the [0,1] interval $R^{3}\to [0,1{]}^{3}$.

$\sigma_{s}=1$: this focuses on a 3 × 3 local window, primarily achieving edge sharpening and texture restoration; $\sigma_{s}=3$: this covers a 7 × 7 region, primarily for medium-range structures; $\sigma_{s}=5$: this ensures consistent fog density through global correlation.

(2) Gaussian Receptive Field Constraint Strategy

The effective range of Gaussian attention is restricted to the local neighborhood via a masking mechanism (15):

(17) $$M_{ij}=\left\{\begin{array}{ll} 1 & if{∥p_{i}-p_{j}∥}_{2}\leq r\\ 0 & \mathrm{otherwise} \end{array}\right.$$

where $p_{i},p_{j}$ are spatial position coordinates and pixel coordinates; the receptive field radius r is scale-adaptive; $M_{ij}=1$ indicates that position i is allowed to focus on position j, conforming to the locality principle; and $M_{ij}=0$ indicates that distant irrelevant regions are masked, reducing the computational redundancy and noise interference.

An innovative dynamic radius adjustment strategy is introduced. In high-entropy regions, where textures are complex, the radius is expanded to capture more context; in low-entropy regions, which are flat areas, the radius is reduced to minimize redundant computations:

(18) $$r=3\sigma_{s}(1+\alpha \cdot Entropy\left(Q_{local}\right))$$

where α is the entropy adjustment intensity coefficient, which controls the influence of local texture complexity on the radius, and is a learnable parameter through Sigmoid activation obtained by dividing into local blocks through the unfold operation; $Q_{local}$ is the local query feature block centered at the current position, $R^{B\times h\times 3\times 3\times d}$; $Entropy\left(Q_{local}\right)$ is the information entropy of local features, which measures the texture complexity,$R^{B\times h\times H\times W}$; the formula to calculate this is $-\sum_{k=1}^{9}\;p_{k}logp_{k}$, $p_{k}=Softmax\left(Q_{\mathrm{local}}^{\left(k\right)}\right)$.

#### 2.2.4. Cauchy–Gauss Branch Dynamic Fusion Module

(1) Hybrid Coefficient Generation

The innovative fog concentration-aware weight prediction mechanism is as follows:

(19) $$\lambda =\sigma ({Conv}_{3\times 3}\;(ReLU\;\left({Conv}_{1\times 1}\;\left(\left[F_{max};F_{avg}\right]\right)\right)))$$

where $F_{max}$ and $F_{avg}$ are the maximum/average pooling features of the input feature map F (along the spatial dimension), mathematically expressed as $R^{C\times H\times W};\;{Conv}_{1\times 1}\;is\;1\times 1$ convolution, with dimension reduction and fusion of global–local features, with mathematical form $R^{2C\times H\times W}\to R^{C\times H\times W};\;ReLU$ is a nonlinear activation function that enhances feature separability, with mathematical form $R^{C\times H\times W}\to R^{C\times H\times W}$; ${Conv}_{3\times 3}$ is 3×3 convolution that captures spatial context relationships, mathematically expressed as $R^{C\times H\times W}\to R^{1\times H\times W};\;\sigma (\cdot )$ is the Sigmoid function activation, which constrains the output to probability weights, and is mathematically expressed as Sigmoid $R^{H\times W}\to [0,1{]}^{H\times W}$.

(2) Gradient problem analysis of mixed operations

Gradient distribution for mixed operations:

(20) $$\frac{\partial L}{\partial \lambda }=\left({\mathrm{Attention}}_{\mathrm{Cauchy}}-{\mathrm{Attention}}_{\mathrm{Gauss}}\right)\cdot \frac{\partial L}{\partial \mathrm{Output}}$$

where $\frac{\partial L}{\partial \lambda }$ is the gradient of the dynamic weight λ, guiding its update direction, with the mathematical form $R^{B\times H\times W}$; ${\mathrm{Attention}}_{\mathrm{Cauchy}}$ is the output of the Cauchy attention branch, which excels at modeling long-range fog concentration dependencies, with the mathematical form $R^{B\times C\times H\times W};{\mathrm{Attention}}_{\mathrm{Gauss}}$ is the output of the Gaussian attention branch, which excels at capturing local details, and is mathematically expressed as $R^{B\times C\times H\times W}$; $\frac{\partial L}{\partial \mathrm{Output}}$ is the gradient of the downstream task (such as dehazing loss) on the mixed output and is mathematically expressed as $R^{B\times C\times H\times W}$ and obtained through backpropagation of the loss function.

When the Cauchy branch performs better than the Gaussian branch, ${(\mathrm{Attention}}_{\mathrm{Cauchy}}$ − ${\mathrm{Attention}}_{\mathrm{Gauss}}$) > 0, the forward gradient increases λ to enhance the contribution of the Cauchy branch; otherwise, λ is reduced to favor the Gaussian branch.

To control gradient competition, the Cauchy branch is prevented from being blocked by λ and suppressing the Gaussian branch, and to improve the training stability, a new method is proposed to prevent λ from over-competing through the stop-gradient strategy.

(21) $$\frac{\partial L}{\partial \lambda }=\left({\mathrm{sg}\;(\mathrm{Attention}}_{\mathrm{Cauchy}})-{\mathrm{Attention}}_{\mathrm{Gauss}}\right)\cdot \frac{\partial L}{\partial \mathrm{Output}}$$

where sg (x)=x is the forward propagation, $\frac{\partial sg(x)}{\partial x}=0$ is the backward propagation, the input and output are completely the same during forward propagation, and no values are changed. During backward propagation, the gradient is forced to zero, and the upstream gradient is truncated here.

#### 2.2.5. Operating Network Design

The mixed distribution attention mechanism combines the advantages of the Cauchy distribution and the Gaussian distribution to optimize the processing of input feature maps $X\in R^{B\times C\times H\times W}$. The specific process is as follows:

1. Dual-branch parallel processing:

The Cauchy branch uses a linearization approximation method to convert the nonlinear Cauchy distribution into a piecewise linear representation through Taylor expansion, significantly reducing the computational complexity. Simultaneously, gradient constraints are introduced to ensure numerical stability during backpropagation and to avoid gradient explosion.

2. The Gaussian branch:

Extracts local features using multi-scale convolution kernels (such as 3 × 3, 5 × 5, 7 × 7), followed by batch normalization layers for each scale branch to satisfy local receptive field constraints. The receptive field is expanded without increasing the number of parameters using dilated convolution. The dynamic fusion mechanism is as follows:

A lightweight gated network is designed to generate fusion weights λ∈[0,1], with the input being the element-wise product of the two-branch features. Dual attention (channel + spatial attention) optimizes the weight distribution, with the formula $\lambda =\sigma \left({Conv}_{3\times 3}\;\left(ReLU\;\left({Conv}_{1\times 1}\;\left(\left[F_{max};F_{avg}\right]\right)\right)\right)\right)$.

A gradient clipping strategy is introduced to constrain the gradient norm in the fusion stage to not exceed the threshold τ, and projection gradient descent is used to ensure training stability.

3. Feature reconstruction output:

The final feature ${\mathrm{Attention}}_{\mathrm{hybrid}}=\lambda \cdot {\mathrm{Attention}}_{\mathrm{Cauchy}}+(1-\lambda )\cdot {\mathrm{Attention}}_{\mathrm{Gauss}}$ is output as $R\in R^{B\times C\times H\times W}$ after residual connection and layerNorm.

### 2.3. Theory and Implementation of Tree-like Multi-Path Coding Structure

#### 2.3.1. Principles of Bio-Inspired Design

**Figure 2:**
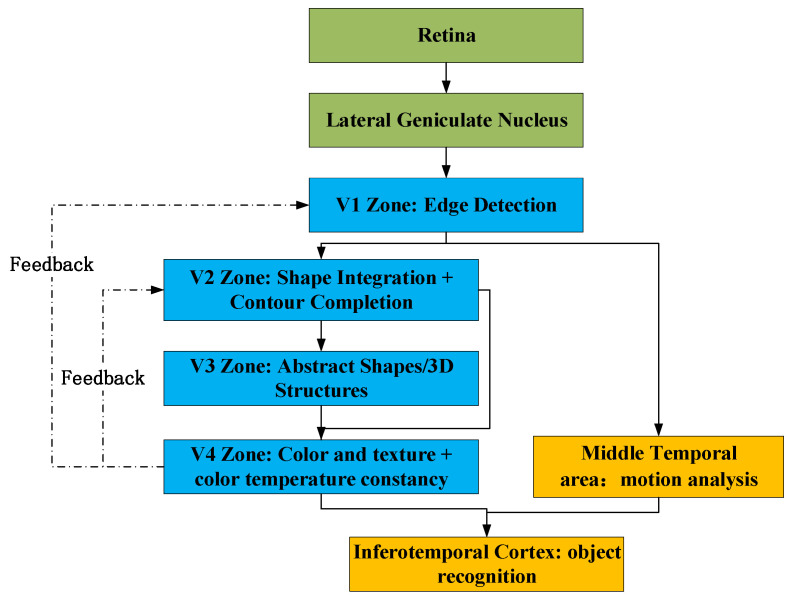
Visual cortex target recognition mechanism diagram.

Inspired by the hierarchical processing mechanism of the primate visual cortex V1–V4 regions [22], as shown in Figure 2, visual cortex target recognition mechanism diagram., these are divided into V1 for edge detection, V2 for shape integration, V3 for shape abstraction, and V4 for color and texture synthesis.

**Figure 3:**
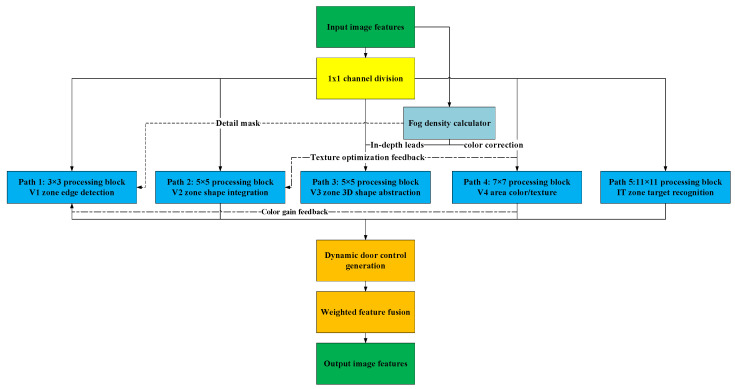
Diagram of the innovative parallel multi-scale processing principle.

Based on the different functions of the V1, V2, V3, and V4 regions of the primate visual cortex, as shown in Figure 3, innovation lies in the following aspects: 1. Parallel multi-scale processing: each path corresponds to different receptive fields (3 × 3 to 11 × 11); 2. Heterogeneous feature extraction: each path independently learns the prior distribution of fog concentration; 3. Dynamic feature fusion: the brain’s attention selection mechanism is simulated.

The multi-pathway collaboration mechanism includes Path 1 (3 × 3 receptive field), which corresponds to the V1 region, extracts local fog line edges, and performs edge detection; Path 2 (5 × 5 receptive field), which corresponds to the V2 region, integrates medium-scale fog cluster structures, and completes shape integration; Path 3 (5 × 5 receptive field), which corresponds to the V3 region and performs 3D shape abstraction; Path 4 (7 × 7 receptive field), which performs color and texture acquisition; and Path 5 (11 × 11 receptive field), which corresponds to the IT region, captures cross-regional semantic associations, and completes object recognition.

#### 2.3.2. Innovative Cauchy Deformation Bimodal Convolution

Adaptive path deformation parameters are based on different functional paths:

(22) $$\Delta p_{k}=\underset{\mathrm{Narrow}\;\mathrm{Peak}}{\underbrace{\gamma_{k}\cdot tanh\;\left(\frac{\pi U_{1}}{\tau }\right)}}+\underset{\mathrm{Wide}\;\mathrm{Peak}\;+\;\mathrm{Noise}}{\underbrace{\eta_{k}\cdot \left[tan\;\left(\pi \left(U_{2}-0.5\right)\right)+\beta \cdot N\;(0,1)\right]}}$$

where $\gamma_{k}=\gamma_{0}+k\cdot \Delta \gamma$ controls the deformation intensity of the narrow peak; $U_{1}\sim Uniform(0,1$) is a random variable that generates the base deformation field; the τ hyperparameter is 0.3~0.5; tanh(·) is a hyperbolic tangent activation function [23] with parameter range [−1, 1]; $\eta_{k}=\eta_{0}\cdot e^{k\lambda }$ is a learnable parameter controlling the wide-peak deformation intensity; $U_{2}\sim Uniform(0,1$) is a random variable; tan(·) is periodic function, (−∞,+∞), generating unbounded deformation (actually constrained to $\pm 3\eta_{k}$); β is a hyperparameter, with a value of 0.05~0.15, and is used to control noise; N(0,1) is a random variable with a standard normal distribution.

A path-adaptive mechanism is adopted: In path 1 (3 × 3 receptive field), corresponding to the V1 region, a narrow peak dominant strategy is used with $\gamma_{k}$ and $\eta_{k}$ values of 0.10 and 0.50, and the τ value is 0.30, each primarily achieving edge detection. In Paths 2 and 3 (5 × 5 receptive field), corresponding to the V2 and V3 regions, a dual-peak balanced strategy is adopted. The V2 regions have $\gamma_{k}$ and $\eta_{k}$ values of 0.15 and 0.67, and the τ value is 0.40; the V3 regions have $\gamma_{k}$ and $\eta_{k}$ values of 0.175 and 0.88, and a τ value of 0.40. Each primarily achieves shape integration. In Path 4 (7 × 7 receptive field), corresponding to the V4 region, a wide-peak dominant strategy is adopted, with $\gamma_{k}$ and $\eta_{k}$ values of 0.20 and 0.90, and a τ value of 0.45, respectively, primarily achieving region fusion. Path 5 (11 × 11 receptive field) corresponds to the IT region, using a wide peak and noise as the dominant peaks, with $\gamma_{k}$ and $\eta_{k}$ values of 0.25 and 1.20, and a τ value of 0.50, respectively, to complete target recognition.

#### 2.3.3. Dynamic Gate Fusion

(1) Fog concentration estimation module.

A lightweight fog concentration estimator is constructed as follows:

(23) $$M_{\mathrm{haze}}=Sigmoid\left({Conv}_{3\times 3}\left(ReLU\left({Conv}_{1\times 1}\left(I_{\mathrm{in}}\right)\right)\right)\right)$$

where $I_{in}\in R^{B\times C\times H\times W}$ is the input image; $M_{\mathrm{haze}}\in [0,1{]}^{H\times W}$ is the output resolution, consistent with the input (H×W×1).

The depth cue extraction formula is as follows:

(24) $$d(x)=-\frac{1}{\beta }log\;\left(1-M_{\mathrm{haze}}\;(x)\right)$$

where β is the atmospheric scattering coefficient (default 0.8); d(x) is the depth map normalized to [0, 1].

The color correction formula is as follows:

(25) $$I_{\mathrm{corrected}}^{c}(x)={\left(I_{\mathrm{in}}\;(x)\cdot \left[1+\alpha \cdot \left(1-M_{\mathrm{haze}}\;(x)\right)\right]\right)}^{1/\gamma }$$

where c∈{R,G,B} is the color channel; α is the gain intensity, with a default of 1.5, and is set to 2.0 for the blue channel; γ is the Gamma correction parameter, with a default of 2.2.

The detail mask formula is as follows:

(26) $$D_{\mathrm{detail}}=Sobel\left(I_{in}\right)⊙\left(1-M_{\mathrm{haze}}\right)+\lambda \cdot TV\left(M_{\mathrm{haze}}\right)$$

where Sobel is the edge detection operator; TV is the total variation regularization term; λ is 0.1 by default.

(2) Gate coefficient generation.

Path weight is calculated as follows:

(27) $$g_{k}=\frac{e^{w_{k}^{T}M_{haze}}}{\sum_{i=0}^{N-1}\;e^{w_{i}^{T}M_{haze}}}\cdot \frac{1}{1+e^{-\alpha_{g}\left(k-\beta_{g}M_{haze}\right)}}$$

where $w_{k}$ is a learnable weight vector, with dimensions =H×W, and a spatial weight matrix of path k with an initial value $N(0,\sqrt{2/(H+W)}$) using gradient backpropagation; $\alpha_{g}$ is the slope factor (initial value 1.0), a scalar that can be learned; and $\beta_{g}$ is the fog concentration sensitivity (initial value 0.5), a scalar that can be learned.

#### 2.3.4. Multi-Path Constraint Strategy

(1) Multi-path gradient allocation.

Gradients from each path are weighted by the gate coefficient:

(28) $$\frac{\partial L}{\partial \theta_{k}}=g_{k}\cdot \frac{\partial L}{\partial \;\mathrm{Output}}+\alpha_{\tau }\cdot \frac{\partial g_{k}}{\partial \theta_{k}}$$

where $\alpha_{\tau }=0.1$ is the gradient balancing coefficient.

(2) Orthogonality Constraint Between Paths

A regularization term is added to promote path differentiation:

(29) $$L_{\mathrm{orth}}=\sum_{i<j}\;[\underset{\mathrm{Cosine}\;\mathrm{similarity}\;\mathrm{penalty}}{\underbrace{{\left|\frac{F_{i}^{T}F_{j}}{{\left\|F_{i}\right\|}_{2}{\left\|F_{j}\right\|}_{2}}\right|}^{2}}}+\underset{\mathrm{Gradient}\;\mathrm{correlation}\;\mathrm{penalty}}{\underbrace{0.1\cdot TV\left(\nabla F_{i}\circ \nabla F_{j}\right)}}]$$

where $F_{i}$ is the output feature map of path i and the dimension is $R^{B\times C\times H\times W}$; $F_{j}$ is the output feature map of path *i* and the dimension is $R^{B\times C\times H\times W};\;{\left\|F_{i}\right\|}_{2}$ and ${\left\|F_{j}\right\|}_{2}$ are scalars with L2 norm; ${\left|\frac{F_{i}^{T}F_{j}}{{\left\|F_{i}\right\|}_{2}{\left\|F_{j}\right\|}_{2}}\right|}^{2}$ is the square operation; $\nabla F_{i}$ and $\nabla F_{j}$ are the spatial gradients of the features of paths i and j and the dimension is $R^{B\times C\times H\times W\times 2};\;and\;\circ$ is the Hadamard product (element-wise multiplication).

#### 2.3.5. Detailed Implementation Steps

1. Input Feature Division

The input channels are divided evenly between the paths using 1×1 convolution:

(30) $$I_{ink}=I_{in}\left[:,\frac{kC}{N}:\frac{(k+1)C}{N}\right],k=0,1,2,3,4$$

where $I_{in}$ is the input feature map with dimensions is $R^{B\times C\times H\times W};\;C$ is the total number of input channels; and $I_{ink}$ is the feature slice assigned to the k path.

2. Independent processing of paths

The data processing formula for each path is as follows:

(31) $$F_{k}={PathBlock}_{k}\left(I_{ink}\right)=GroupNorm\left(ShuffleConv\left(CauchyDeformConv\left(I_{ink}\right)\right)\right)$$

where CauchyDeformConv is an innovative Cauchy deformation double-peak convolution function, calculated with Formula (32):

(32) $$CauchyDeformConv\left(I_{ink}\right)=\sum_{i=1}^{N_{k}}\;w_{i}\cdot I_{ink}\left(p_{0}+\Delta p_{i}\right)\cdot \Delta m_{i}$$

where $I_{ink}\left(p_{0}+\Delta p_{i}\right)$ is the eigenvalue of the input feature map $I_{in}$ at $p_{0}+\Delta p_{i}$; $p_{0}$ is the integer coordinate of the current convolution center point in the two-dimensional coordinate (x,y); $\Delta p_{i}$ is the two-dimensional coordinate offset using Formula (22) and the offset of the $I_{in}$ sampling point; $\Delta m_{i}=Sigmoid\left(MLP{\left(I_{ink}\right)}_{{:,p}_{0}}\right)$ is the modulation factor, with an initial value of 1, constrained by the Sigmoid function; $m_{i}\in \{0,1\}$, $m_{i}=\left\{\begin{array}{ll} 1 & \mathrm{if}\;\Delta p_{i}\mathrm{close}\;\mathrm{to}\;+\delta \\ 0 & \mathrm{if}\;\Delta p_{i}\mathrm{close}\;\mathrm{to}\;-\delta \end{array}\right.,\;\delta$ is the double-peak spacing, $w_{i}=Softmax{\left({Conv}_{1\times 1}{\left(I_{ink}\right)}_{{:,p}_{0}}\right)}_{i}$ is a dynamic scalar and is the weight of the i sampling point, $\sum_{i=1}^{N_{k}}\;w_{i}=1$; $N_{k}$ is the total number of sampling points, $N_{k}$ = 9 for a 3 × 3 convolution kernel.

ShuffleConv is a channel shuffle convolution that enhances cross-group information flow, as shown in Formula (33):

(33) $$ShuffleConv(I_{inck})=ReLU(Conv(P(I_{inck};G),K_{H},\mathrm{groups}=G))$$

where $P(I_{inck};\;G$) is the channel shuffle operation (divides C channels into G *groups* and permutes them); $K_{H}$ is the convolution kernel.

GroupNorm is the group normalization function, defined as (34):

(34) $$GroupNorm\left(I_{incsk}\right)=\gamma_{gn}\cdot \frac{I_{incsk}-\mu_{g}}{\sqrt{\sigma_{g}^{2}+\epsilon }}+\beta_{gn}$$

where $\mu_{g}$ and $\sigma_{g}$ are the mean/variance of each group of channels; $\gamma_{gn},\beta_{gn}\in R^{C}$ are learnable affine parameters; and $\epsilon =10^{-5}$ is a numerical stability term.

3. Dynamic feature fusion

The output result is the weighted sum of the features of each path:

(35) $$I_{\mathrm{out}}=\sum_{k=0}^{N_{k}-1}\;g_{k}\cdot {Conv}_{1\times 1}\left(F_{k}\right)$$

where $N_{k}$ is the number of paths plus 1; $F_{k}$ is the output feature of the k path; $g_{k}$ is the dynamically generated weight of the k path.

## 3. Operational Process and Innovative Advantages

### 3.1. Operational Process

The model adopts a U-Net-shaped encoder–decoder structure, achieving multi-scale feature extraction through four-stage downsampling and upsampling. It innovatively introduces the Cauchy distribution into deformation convolution, constructing a three-stage processing workflow: “Cauchy deformation convolution—multi-scale Cauchy–Gaussian attention—tree-like multi-path encoding hybrid distribution fusion.”

**Figure 4:**
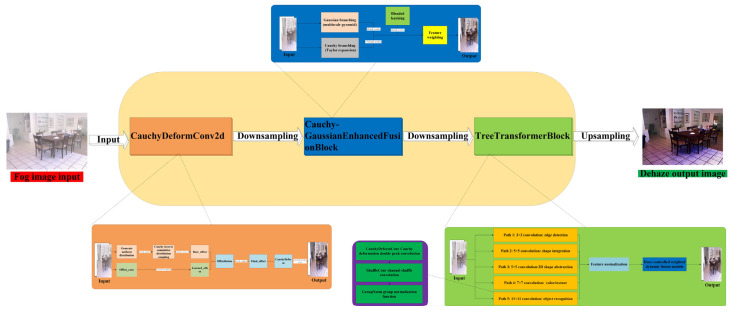
Algorithm execution flowchart.

As shown in Figure 4, the algorithm runs as follows (Algorithm 1):
**Algorithm 1** EnhancedMB-CauchyFormer Pseudocode  I—Input: Image tensor (B, C, H, W),—Apply 3x3 convolution with stride 1 and padding 1, O—Output: Embedded patches (B, embed_dim, H, W) 1: function EnhancedMB-CauchyFormer(I): 2:    x = I // Receive raw image input 3:    x = OverlapPatchEmbed(x) // Initial feature extraction [B, C0, H, W] 4:    // --- Level 1 --- 5:    x1_list = Patch_Embed_stage_Cauchy(x, num_path=N1) // Generate N1 paths [B, C0, H, W] × N1 6:    x1 = EnhancedMHCA_stage(x1_list) + x // Tree-based multi-head attention + residual connection 7:    // --- Level 2 --- 8:    x2 = Downsample(x1) // Downsampling [B, C1, H/2, W/2] 9:    x2_list = Patch_Embed_stage_Cauchy(x2, num_path=N2) 10:   x2 = EnhancedMHCA_stage(x2_list) + x2 11:   // --- Level 3 --- 12:   x3 = Downsample(x2) // Downsampling [B, C2, H/4, W/4] 13:   x3_list = Patch_Embed_stage_Cauchy(x3, num_path=N3) 14:   x3 = EnhancedMHCA_stage(x3_list) + x3 15:   // --- Level 4 (Latent) --- 16:   x4 = Downsample(x3) // Downsampling [B, C3, H/8, W/8] 17:   x4_list = Patch_Embed_stage_Cauchy(x4, num_path=N4) 18:   x4 = EnhancedMHCA_stage(x4_list) + x4 19:   // ======== 4. Decoder Upsampling Path ======== 20:   // --- Level 3 --- 21:   x3_up = Upsample(x4) // Upsampling [B, C2, H/4, W/4] 22:   x3_up = Concat(x3_up, x3) // Concatenate with encoder features 23:   x3_up = ReduceChannels(x3_up) // 1 × 1 convolution for dimension reduction 24:   x3_up_list = Patch_Embed_stage_Cauchy(x3_up, num_path=N3) 25:   x3_up = EnhancedMHCA_stage(x3_up_list) + x3_up 26:   // --- Level 2 --- 27:   x2_up = Upsample(x3_up) // Upsampling [B, C1, H/2, W/2] 28:   x2_up = Concat(x2_up, x2) 29:   x2_up = ReduceChannels(x2_up) 30:   x2_up_list = Patch_Embed_stage_Cauchy(x2_up, num_path=N2) 31:   x2_up = EnhancedMHCA_stage(x2_up_list) + x2_up 32:   // --- Level 1 --- 33:   x1_up = Upsample(x2_up) // Upsampling [B, C0, H, W] 34:   x1_up = Concat(x1_up, x1) 35:   x1_up_list = Patch_Embed_stage_Cauchy(x1_up, num_path=N1) 36:   x1_up = EnhancedMHCA_stage(x1_up_list) + x1_up 37:   // ======== 5. Refinement Stage ======== 38:   x_refine_list = Patch_Embed_stage_Cauchy(x1_up, num_path=N1) 39:   x_refine = EnhancedMHCA_stage(x_refine_list) + x1_up 40:   // ======== 6. Output Processing ======== 41:   if dual_pixel_task: 42:      x_refine = x_refine + SkipConv(x) // Skip connection 43:     O = OutputConv(x_refine) // 3-channel output 44:   else: 45:      O = OutputConv(x_refine) + I // Residual output 46: return O

### 3.2. Methodological Advantages

This network proposes a bio-inspired multi-path defogging architecture, the core algorithm of which consists of three innovative modules. The workflow can be divided into three stages: feature decoupling, multi-scale fusion, and dynamic reconstruction.

#### 3.2.1. Cauchy Deformable Convolution

Cauchy deformable convolution (CDC), as an innovative module of the Enhanced Multi-Branch Cauchy Transformer Network (EnhancedMB-CauchyFormer) algorithm, offers the following significant advantages over traditional deformable convolution (DCN) and standard convolution: 1. In image restoration tasks (such as dehazing and denoising), the displacement of edges and texture regions often suddenly changes. The Cauchy inverse accumulation function can better adapt to such irregular deformations. The double-peak Cauchy inverse accumulation distribution retains the advantages of a single-peak heavy tail while explicitly modeling multi-modal displacement, significantly enhancing the method’s expressive capability in complex deformation scenarios. 2. The heavy-tail characteristic of the Cauchy inverse accumulation distribution function makes it more robust to outliers. 3. An adaptive correction method is used for continuous learning to enhance the algorithm’s adaptability.

#### 3.2.2. Innovative Dual-Distribution Hybrid Mechanism

As described in Section 2.2, by integrating the Cauchy inverse cumulative distribution function and the Gaussian distribution, and automatically adjusting the weights of the two distributions through learnable parameters λ, we propose a differentiable dual-distribution sampling mechanism that combines mixed probability distributions with feature fusion for the first time, and derive a Cauchy–Gaussian hybrid gradient formula suitable for deep learning.

A lightweight fuzzy detection network (only two convolutional layers) is designed to achieve feature enhancement with degraded perception. Gradient stabilization techniques are developed to address the numerical instability of the Cauchy distribution during backpropagation. Compared with other attention fusion methods, this approach offers the best robustness. The fuzzy region is explicitly modeled, resulting in strong anti-fuzzy performance. Training is stable and does not require fine-tuning, as the system automatically learns to balance parameters, and the computational overhead is relatively small.

#### 3.2.3. Innovative Tree-Based Self-Attention Model Block

For parallel branch processing, the total computation of four branches is only one-fourth that of the standard self-attention model; window attention locality avoids global attention matrix computation; blur mask guidance automatically enhances high-frequency features in motion blur regions; and thick-tail distribution robustness enables Cauchy branches to effectively suppress blur artifacts.

## 4. Experimental Setup and Result Analysis

### 4.1. Algorithm Setup

We selected the Dark Channel Prior (DCP) [1], Improved All-in-One Dehazing Network (AOD-Net) [24,25], Multi-Scale Boosted Dehazing Network with Dynamic Feature Fusion (MSBDN-DFF) [26], Feature Fusion Attention Network (FFA-Net) [27], Revitalizing Convolutional Network (ConvIR) [28], Frequency Domain Assistance and Detailed Brightness Information Guidance (FIGD-Net) [15], Multi-Branch Taylor Transformer V2 (MB-TaylorFormerV2) [16,17], and Enhanced Multi-Branch Cauchy Transformer Network (EnhancedMB-CauchyFormer) for the comparative analysis. The input parameters of the algorithms were uniformly configured.

Model Architecture: EnhancedMB-CauchyFormer, number of parameters: 15.8 M; computational complexity: 96.8GFlops; employs 5-branch dynamic weight allocation + multi-scale Cauchy feature fusion. We set the initial learning rate to 2 × 10^−4^ and gradually reduced it to 1 × 10^−6^ using sine annealing [29].

Training Configuration Data Augmentation: random cropping to 256 × 256 pixels (overlap rate ≥ 30%); probabilistic horizontal flipping (*p* = 0.5); color space perturbation (ΔHSV∈ [0.1, 0.3]).

### 4.2. Experiment on Synthetic Blur and Real Blur Images

**Figure 5:**
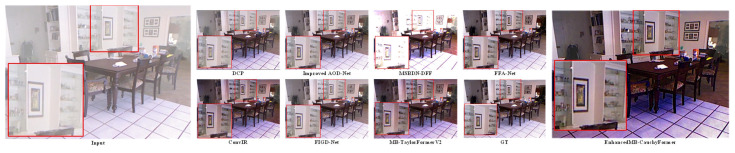
SOTS-Indoor image comparison experiment. The red large frame is enlarged by 1 times compared to the red small frame.

**Figure 6:**
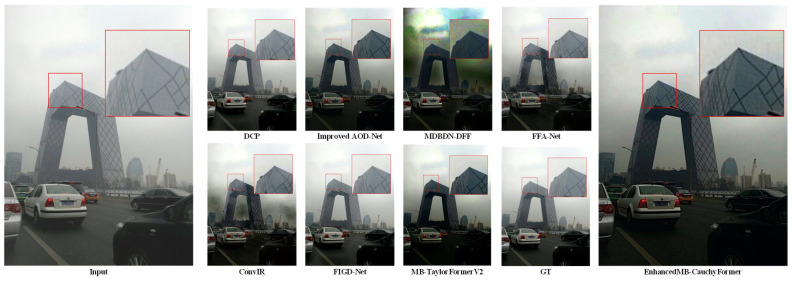
SOTS-Outdoor image comparison experiment. The red large frame is enlarged by 4 times compared to the red small frame.

**Figure 7:**
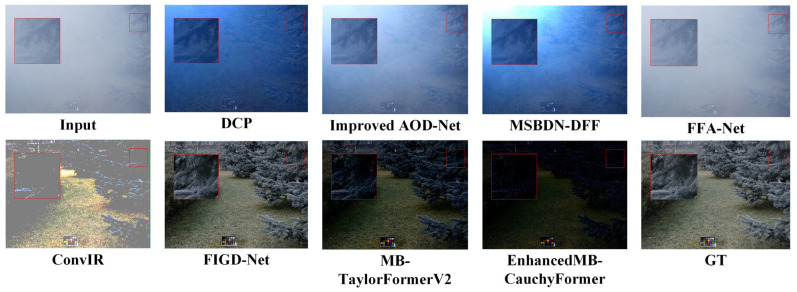
O-HAZE image comparison experiment. The red large frame is enlarged by 4 times compared to the red small frame.

**Figure 8:**
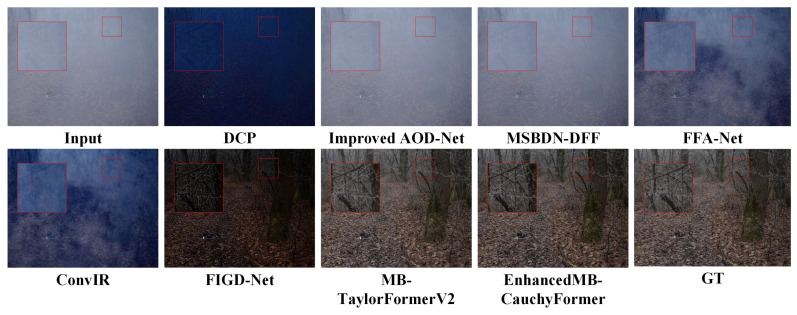
Dense-haze image comparison experiment. The red large frame is enlarged by 4 times compared to the red small frame.

Using the data set in Table 1, the test results are shown in Table 2. In the RESIDE benchmark test, the EnhancedMB-CauchyFormer demonstrated excellent performance improvements: its Cauchy–Gaussian hybrid attention mechanism maintained robustness even under extreme fog concentrations (Fog concentration >0.8). In synthetic haze scenes, the EnhancedMB-CauchyFormer achieved a peak signal-to-noise ratio (PSNR) of 45.12 dB and a Structural Similarity Index Measure (SSIM) of 0.997 with 15.80 million parameters in indoor scenes (SOTS-Indoor), representing a 5.27% improvement in the PSNR compared to that with MB-TaylorFormerV2, as shown in the experimental comparison in Figure 5, where GT denotes the original image. This demonstrates the modeling advantages of the Cauchy distribution for synthetic haze, enabling better image restoration. In outdoor scenes (SOTS-Outdoor) under complex weather conditions, the adaptability to outdoor scenes was improved. EnhancedMB-CauchyFormer achieved a PSNR of 39.55 dB, surpassing that of MB-TaylorFormerV2 (39.25 dB) by 0.25 dB, as shown in Figure 6, demonstrating robustness to changes in outdoor lighting. In real haze scenes, on the O-HAZE dataset, the PSNR value decreased by 1.14, and the SSIM value decreased by 0.05, as shown in the experimental comparison in Figure 7. This dataset maintains its structural similarity advantage. In the Dense-Haze dataset, the PSNR improved by 0.12 dB, representing a 0.68% increase, as shown in Figure 8, indicating that the proposed method has superior structural restoration capabilities in high-concentration haze compared to those of existing techniques. The lowest performance-to-computation ratio (PSNR/MACs) among the state-of-the-art methods was 0.466, outperforming MB-TaylorFormerV2’s 0.498, demonstrating superior computational efficiency compared to that of similar attention-based models.

### 4.3. Ablation Studies

All of the models were trained on the SOTS-Indoor dataset, with an input consisting of randomly cropped 256 × 256 pixel patches, a batch size of 32, and the Adam with Weight Decay optimizer ($\beta_{1}=0.9,\beta_{2}=0.999$). The base learning rate was set to 2 × 10^−4^ and was decayed to 1 × 10^−6^ over 500 epochs using sine annealing scheduling. Based on the EnhancedMB-CauchyFormer architecture, the effects of the Cauchy variable convolution layer, hybrid attention mechanism, and tree-like multi-branch architecture were analyzed sequentially, and the synergistic effects of the modules were examined.

#### 4.3.1. Cauchy Deformable Convolution Layer

As can be seen from Table 3, the Cauchy deformable convolutions achieved a further gain of 0.25 dB over the Gaussian deformable convolutions, and their depth-separable Cauchy convolutions more accurately modeled the physical distribution characteristics of fog.

#### 4.3.2. Cauchy–Gauss Hybrid Attention Mechanism

As can be seen from Table 4, the Cauchy–Gauss kernel approximation, compared to the standard attention mechanism, improves the PSNR by 1.88 dB and the SSIM from 0.991 to 0.995, demonstrating its superior structural preservation capability. The Cauchy kernel approximation error, achieved through a third-order Taylor expansion, is only 0.021, significantly outperforming linear attention (0.148), making it closer to the ideal attention concentration modeling capability. The long-range modeling of the Cauchy distribution’s long-tail characteristics achieves a cross-image block dependency strength of 0.92, which is 2.9 times that of linear attention, making it particularly beneficial for global consistency restoration in heavily fogged regions.

#### 4.3.3. Tree-like Multi-Path Branch Architecture

As shown in Table 5, the effectiveness of multi-path processing and fusion is demonstrated. The five branches represent the five paths of the tree, and adaptive feature selection is achieved through a gating mechanism. The coupling of edge detection (L1) and shape integration (L2) improves the contour PSNR by 2.3% (44.98 dB vs. 43.95 dB), validating the guiding role of mid-frequency information in edge restoration. At the color and texture recognition (L4) turning point, after introducing color and texture paths, the SSIM increased from 0.994 to 0.995, indicating that color consistency can mitigate color bias issues.

#### 4.3.4. Module Synergy Analysis

Table 6 shows a comparison of the performance of the four image processing models, illustrating the gradual improvements achieved through algorithm optimization. The baseline model (standard convolution + attention) achieves a PSNR of 40.71 dB, an SSIM of 0.991, and a computational load of just 3.2 GFLOPs, offering a lightweight advantage. Cauchy–Fourier transform introduces a frequency domain analysis, improving the PSNR by 1.16 dB (a relative increase of 26.3%) while increasing the computational complexity to 7.8 GFLOPs. This significantly enhances the defogging performance while maintaining low computational costs. The hybrid distribution attention combining Cauchy narrow peaks and Gaussian wide peaks achieves a PSNR reaching 42.59 dB (an increase of 1.88 dB) and an SSIM of 0.995, enhancing the adaptability to complex fog distributions. The final tree-based multi-branch complete model performs optimally, achieving a PSNR of 45.12 dB (an increase of 4.41 dB, or a 57.4% relative improvement) and an SSIM of 0.996, demonstrating robust multi-scale feature fusion capabilities. Although the computational load increased to 96.8 GFLOPs, the model achieved a strong improvement in accuracy and is suitable for high-precision defogging tasks. Overall, the algorithm has been progressively optimized in terms of the PSNR, SSIM, and adaptability, while maintaining a reasonable balance between computational efficiency and performance improvements.

### 4.4. Real Target Recognition and Verification

**Figure 9:**
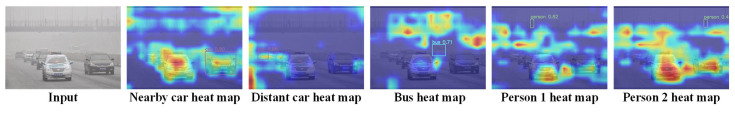
Different target heat maps.

As shown in the target recognition heat map in Figure 9, EnhancedMB-CauchyFormer significantly improves the explainability of target recognition: the red area (0.8–1.0) accurately covers key decision areas such as the front of cars, bus doors, and the upper bodies of pedestrians; the yellow area (0.5–0.8) effectively captures auxiliary features such as wheels, windows, and the limbs of pedestrians; and the background suppression (blue < 0.5) reduces false positives.

**Figure 10:**
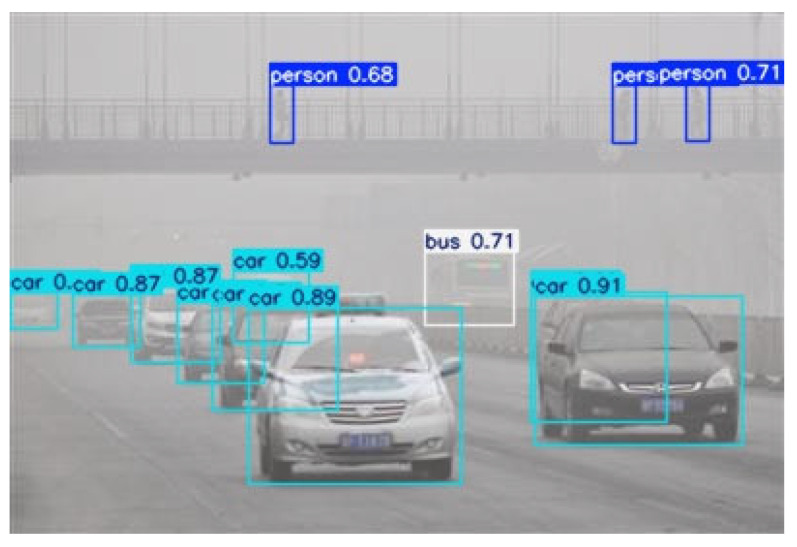
Real target recognition and probability map.

As shown in Figure 10, for image recognition under real foggy weather conditions, the algorithm recognizes small cars, pedestrians, and buses in foggy weather conditions. It accurately identifies small pedestrian targets on bridges and precisely distinguishes between small cars and buses, demonstrating the algorithm’s advantage of multi-branch fusion to adapt to targets of different scales. The Cauchy variable convolution enhances edges and small features as well as long-tail features. Dynamic weight learning automatically adapts to simple or complex scenes, and the verification results align with the research findings.

## 5. Conclusions

In this paper, we propose a novel defogging algorithm inspired by a bio-inspired multi-path defogging architecture, which addresses the performance bottlenecks of traditional methods in dense fog edges and strong illumination fog lines through Cauchy inverse cumulative function variable convolution and multi-scale fusion. Based on an innovative multi-peak Cauchy inverse cumulative distribution offset generator, the algorithm precisely models sudden changes in fog concentration using heavy-tailed characteristics; a hybrid Cauchy–Gaussian module dynamically balances the representation of smooth regions and edge details; and a tree-based multi-path model identification achieves adaptive local–global feature fusion. The algorithm’s superiority was validated through comparisons across various datasets, and its effectiveness was demonstrated through target recognition tests in real-world dense fog conditions.

## Figures and Tables

**Table 1:** Dataset configuration table.

Dataset	Training Sample Count	Test Sample Count	Fog Concentration Distribution	Evaluation Subset
RESIDE-ITS [30]	13,990	500	Low → high (indoor)	SOTS
RESIDE-OTS [30]	313,950	500	Uniform (outdoor)	SOTS
O-HAZE [31]	40	5	Medium (real scene)	Last 5
Dense-HAZE [32]	50	5	Extremely high (heavy fog)	Last 5

**Table 2:** Quantitative comparison of different methods in fog removal benchmark testing.

Method	SOTS-Indoor	SOTS-Outdoor	O-HAZE	Dense-Haze	Computational Efficiency
-	PSNR ↑ SSIM ↑	PSNR ↑ SSIM ↑	PSNR ↑ SSIM ↑	PSNR ↑ SSIM ↑	Params (M) MACs (G)
Based on physical models	-	-	-	-	-
DCP [1]	16.62 0.818	19.13 0.815	16.78 0.653	12.72 0.442	- 0.78
Improved AOD-Net [24,25]	24.61 0.854	22.356 0.934	20.577 0.723	13.59 0.544	0.025 2.3
MSBDN-DFF [26]	34.29 0.983	33.65 0.970	24.87 0.758	16.34 0.638	31.45 43.82
Self-attention model	-	-	-	-	-
FFA-Net [27]	36.39 0.989	33.57 0.984	22.12 0.770	15.70 0.549	4.46 287.8
ConvIR [28]	41.53 0.994	37.95 0.990	25.36 0.784	16.86 0.600	5.53 42.1
SOTA	-	-	-	-	-
FIGD-Net [15]	41.11 0.996	38.19 0.9992	**26.12 0.805**	17.71 0.608	124.24 70.76
MB-TaylorFormerV2 [16,17]	42.86 0.995	39.25 0.992	25.43 0.792	16.95 0.621	7.29 86.0
Ours (SOTA breakthrough)	-	-	-	-	-
EnhancedMB-CauchyFormer	**45.12 0.997**	**39.55 0.994**	24.98 0.800	**17.83 0.584**	15.80 96.8

Here, “-” indicates that the result is not available. The best and second best results are highlighted in bold and underlined, respectively. The core meaning of the upward arrow (↑) Indicates that “the higher the value, the better the performance”: The arrow ↑ clearly indicates that the larger the value of the indicator (such as PSNR, SSIM), the better the algorithm effect.

**Table 3:** Comparison of different convolution layer strategies.

Method	PSNR (dB)	SSIM	Parameters (M)	MACs (G)
Standard convolution	40.71	0.991	1.8	3.2
Gaussian deformable convolution	41.62	0.992	2.5	5.1
Cauchy deformable convolution	41.87	0.993	2.7	7.8

**Table 4:** Comparison of the approximation error and performance of different attention mechanisms.

Attention Type	PSNR (dB)	SSIM	Approximation	Long-Range Dependency Strength
Standard attention	40.71	0.991	0	1.0
Linear attention	36.12	0.973	0.148	0.32
Gaussian kernel approximation	40.05	0.989	0.087	0.75
Cauchy–Gaussian Kernel Approximation	42.59	0.995	0.021	0.92

**Table 5:** Progressive performance of branch combinations.

Activate Branch	PSNR (dB)	SSIM	Main Domain
L1	43.95	0.994	Edge texture
L1 + L2 + L3	44.98	0.993	Contour shape integration
L1 + L2 + L3 + L4	45.02	0.995	Global color/texture shape modeling
L1 + L2 + L3 + L4 + L5	45.08	0.996	Target recognition
Full branch + dynamic gate control	45.12	0.996	Cross-scale feature fusion

**Table 6:** Module synergy analysis.

Module Combination	PSNR (dB)	SSIM	ΔPSNR vs. Baseline	Contribution Decomposition	MACs (G)
Baseline (standard convolution+ attention)	40.71	0.991	—	—	3.2
Cauchy–Fourier transform	41.87	0.993	+1.16	26.3%	7.8
Mixed distribution attention	42.59	0.995	+1.88	16.3%	58.8
Tree-like multi-branching (complete model)	45.12	0.996	+4.41	57.4%	96.8

## Data Availability

The data are contained within the article.

## Abbreviations

The following abbreviations are used in this manuscript:

ICDF	Inverse Cumulative Distribution Function
CNN	Convolutional Neural Network
MB-TaylorFormer	Multi-Branch Taylor Transformer
DCP	Dark Channel Prior
AOD-Net	All-in-One Dehazing Network
MSBDN	Multi-Scale Boosted Dehazing Network
FFA-Net	Feature Fusion Attention Network
Restormer	Restoration Transformer
Dehamer	Deep Hybrid Atmospheric-scattering Model Guided Network
EnhancedMB-CauchyFormer	Enhanced Multi-Branch Cauchy Transformer Network
PSNR	Peak Signal-to-Noise Ratio
SSIM	Structural Similarity Index Measure
